# Phytochemical analysis and anti-microbial activities of *Artemisia* spp. and rapid isolation methods of artemisinin

**DOI:** 10.1186/s13568-022-01346-5

**Published:** 2022-02-12

**Authors:** Seid Mohammed, Aman Dekabo, Tilahun Hailu

**Affiliations:** 1 Present Address: Adama Science and Technology University, Oromia, Ethiopia; 2 Department of Applied Chemistry, School of Applied Natural Science, Oromia, Ethiopia

**Keywords:** *Artemisia*, Anti-bacteria, Anti-fungi, Artemisinin, Sesquiterpene lactone, Dibydro-epideoxyartannrrin B

## Abstract

Artemisia species are important traditional medicinal plants of Ethiopia which are used for the treatment of infection and non-infection health problems. The genus *Artemisia* (Astraceae) consists of about 500 species worldwide. In this study, the main aim was to investigate chemical components of *Artemisia* spp. (*A. abyssinica,* A. *absinthium* and A. *annua*) and evaluate their antimicrobial activities against bacterial strains. The results indicated that the crude extract of these plants was effective against selected bacterial strains. Here we isolated the well-known antimalarial drug artemisinin (7 mg, 0.004%) from *Artemisia annua* leaves using a rapid *n*-hexane fractionation method*.* The *n-*hexane extract of *A. abyssinica,* ethyl acetate extract of *A. absinthium* and *n*-hexane of *A*. *annua* showed varying degrees of inhibiting effect against bacterial strains such as S*taphylococcus aureus* ATCC 25923^ T^, *Salmonella enteritidis* ATCC13076^T^, *Klebsiella pneumoniae* ATCC1053^T^, *boydii* ATCC1233^T^, *Escherichia coli* ATCC 25922^ T^, hospital acquired *Acinetobacter baumannii.* The ethyl acetate extract of *Artemisia absinthium* (A.ab_e_) showed the maximum inhibiting effect (35 mm) against *A. baumannii*. The minimum zone of inhibition (< 3 mm) was recorded for test extract of A.ap against *Klebsiella pneumoniae* ATCC1053^T^. Ethyl acetate extract of *A, absinthium* (A.abe) was more effective against these selected bacterial strains and the zone of inhibition ranged from 5 to 35 mm. The minimum inhibition zone (8 mm) was detected against *S. typhimurium* ATCC 13311^ T^ for both A.ac and n-hexane- EtOAc fraction (8:2) of *A, abyssinica*. The maximum zone of inhibition (25 mm) for fraction (A.ach F4) of *A, abyssinica* obtained by column chromatography was recorded against *S. pyogen ATCC* 19,615. However, there was no zone of inhibition detected for *boydii* ATCC1233^T^ due to these test extracts. Significant variations (P ≥ 0.05) were observed between all test extracts of these medicinal plants at 95% of confidence intervals. In this study, clear zones of inhibition were detected for the positive control (standard impregnated disks). Based on our results we recommend that various species of *Artemisia* spp. seem to have the potential for in-depth investigation for various antimicrobial activities that assisting the effort in searching for antimicrobial lead compounds.

**Supplementary Information:**

The online version contains supplementary material available at 10.1186/s13568-022-01346-5.

## Introduction

The practice of exploiting natural products, the flora and fauna, to alleviate pain and to cure diseases of humans as well as domestic animals is as old as the history of human civilization. Nowadays, the uses of traditional medicinal plants for treatment of some diseases are increasing throughout the world. For instance, in Africa alone, nearly 80% of the populations use medicinal plants for their primary health care. Similarly, in countries like China about 30–35% traditional herbal plants have been employed as a medicinal plants (World Health Organization 2002). The *Artemisia* spp. have been also considered as bitter herbs or shrubs in some countries such as Asiatic Steppes, South Africa, South America and the United States (Rana et al. 2013). It has been reported that *Artemisia* spp, is the largest genera of Asteraceae that is widely distributed in the northern hemisphere. These plants are aromatic with essential oils used as perfumery and medicinal plants (Kalemba et al. 2002).

In Ethiopia, these medicinal plants have a unique and diverse botanical heritage with diverse topography, vegetables, and various climatic conditions (Asefa et al. 2020). For instance, these *Artemisia* species have been reported in Asia, Europe, and North America. These medicinal plants are also found in other different countries. These medicinal plants consist of about 500 species. Some of them are small herbs and belong to the Asteraceae family (Bora and Sharma 2011; Willcox 2009).

It has been recognized that certain traditional medicinal plants have been employed and act as a first-line difference for microbial pathogens. These medicinal plants have been commonly used for treatment of high fever that occurred due to malaria in countries such as Ghana, Mali, Nigeria and Zambia (World Health Organization 2002). It has been confirmed that the *Artemisia* spp have contained essential oils that is able to inhibit certain pathogenic bacterial growth such as *Staphylococcus aureus*, and *Staphylococcus epidermidis*. For instance, *Artemisia asiatica* Nakai contains essential oils such as 1,8-cineole, selin-11-en-4_-ol and monoterpene. These essential oils are used to inhibit *Bacillus subtilis, Escherichia coli, Pseudomonas aeruginosa* and *S. aureus* (Kalemba et al. 2002)*,.* Certain fungi species such as *Aspergillus niger, Candida albicans*, *Cryptococcus neoformans*, *Microsporum canis,* and *M. gypseum*, *Trichophyton rubrum*, have also been found to be inhibited by essential oil obtained from *Artemisia s*pp in Canada (Lopes-Lutz et al. 2008). It has been further stated that the essential oils of *A. asiatica* Nakai are employed to inhibit *Aspergillus fumigatus*, *Candida albicans,* and *Rhodotorula rubra* (Kalemba et al. 2002)*.*

Recently however, the use of essential oils and other chemical extracts from plants gained importance due to the increase of their application in pharmaceutical products. They have shown biological activities such as antifungal, antimicrobial, antioxidant, and antiviral activities. In addition some of them have been used in cancer treatment (Abdelwahab et al. 2010; Sylvestre et al. 2006).

*Artemisia species*, widespread throughout the world, are one of the widely known plants in folk and modern medicine preparations. They are frequently used for the treatment of diseases such as malaria, hepatitis, cancer, inflammation and infections by fungi, bacteria and viruses. Extensive studies of the chemical components of *Artemisia species* have led to the identification of many inhibitory compounds. For instance, Artemisinin is a choice inhibitory compound which is used as a choice of drug for the treatment of malaria. It is obtained from *A. annua* and some microbial sources as a result of genetic engineering. Artemisinin, is a best known inhibitory compound for treatment of *Trypanosoma brucei brucei* with an IC_50_ value of 35.91 µg/ml and with a selectivity index of 2.44 (Mannan et al. 2010; Nibret and Wink 2010). This inhibitory compound, Artemisinin may be used to heal or treat a certain disease. For instance, It has been documented that *Artemisia* spp*.* are frequently utilized for the treatment certain diseases such as cancer, hepatitis, inflammation, infections due to bacteria, fungi, malaria, and viruses (Willcox 2009). The antifungal activity of certain *Artemisia* species has been reported. For instance, it has been found that *Artemisia* extract compounds such as carvone, and piperitone are used to inhibit *Mucora rouxii* and *Penicillium citrinum* (Chung et al. 2009)*.*

*A. absinthium is* traditionally known as *Ariti* in Ethiopia. This *Artemisia* spp. is typically identified as erect, and perennial herb with 30–60 cm in length (Yineger et al. 2007). The same *Artemisia* species have been reported in western Canada (Lopes-Lutz et al. 2008). These medicinal plants have been cultivated in some parts of Ethiopia for its natural aroma and applied during ritual conditions and also referred to *adbar* (Yineger et al. 2007). However, the same *Artemisia* spp. particularly their essential oil extracts have been employed for inhibiting pathogenic bacterial cells like *Staphylococcus* strains (Lopes-Lutz et al. 2008). Some study have shown that *A.absinthium* extracts have been found to use as remedy for the treatment of malaria and is also employed in combination with other herbs for treating wounds of domestic animals (Yineger et al. 2007).

*A. abyssinica* (known as ‘chikugn’ in Ethiopia) is an erect, annual or short-lived perennial herb, 30–60 cm high. It is quite commonly used in traditional medicine and in rituals. It has been reported for treatment of diseases such as rabies, tonsillitis, gonorrhea, cough, syphilis, and leprosy. The fresh roots of the same herbs are used to treat epilepsy in domestic animals (Geyid et al. 2005; Yineger et al. 2007).

*A.annua* is an erect aromatic annual herb of up to 2 m in height. It is a common weed over large parts of Eastern Europe and Asia, and has become naturalized in North America. It is cultivated on a commercial scale in eastern China, in the Balkans, and more recently in India and Africa (Van Wyk 2011). It is an exotic species introduced ten years ago from abroad and is currently cultivated in southern Ethiopia mainly for its traditional antimalarial “herbal tea” consumption and as a remedy for Hemorrhoid, Asthma, and Common cold. It is a source for the production of artemisinin, a sesquiterpene lactone with anti-malarial effects against susceptible and multi-drug resistant Plasmodium species (Hailu et al. 2013; Nibret and Wink 2010).

The volatile components and essential oils of *A.absinthium, A. abyssinica, A. afra,* and *A. annua* are derived from leaves and aerial parts. Their components have been characterized by using GLC/MS, GC–MS, and GC (Lopes-Lutz et al. 2008; Nibret and Wink 2010). It was also found that dichloromethane extract of *A. absinthium* contains camphor (38.73%) which is a major component of the compound (Nibret and Wink 2010). Some antioxidant such as beta-carotene or linoleate and 2,2-diphenyl-1-picrylhydrazyl (DPPH) radical have also been reported by using GC–MS, and GC for extracts of *A.absinthium* (Lopes-Lutz et al. 2008)*.* Certain natural products or volatile component such as Octa-3,5-diene-2,7-dione,4,5-dihydroxy (*A. abyssinica,* 54.95%), Epoxylinalool (*A. afra*, 29.10%), and Deoxyqinghaosu (*A. annua,* 20.44%) have been obtained from dichloromethane extract for *Artemisia* species (Nibret and Wink 2010)*.*

A large number of medicinal plant consents such as essential oils, terpenes, sesquiterpenes, and alkaloids have been shown to be found in *Artemisia species* associated with antiprotozoal, antimicrobial, and antifungal activities (Valdés et al. 2008). Nevertheless, a considerable amount of research is still needed to explain the curative effect associated with traditional herbal remedies, to identify simple technology that could produce therapeutic agents at low cost for the alleviation of suffering and infectious disease widespread in the world. The wide utilization of the above three *Artemisia species* for various diseases of infectious and non-infectious origins triggered us to investigate their chemical compositions and evaluate their in vitro effects against some group of infectious bacteria.

## Material and methods

### Sample collection

The samples of *A.absinthium* which is locally known as “*Arity*” were collected from around Bale Robe, Indato Gasera (Additional file 1: Fig. S1a) and *A. annua* were obtained from Wondogenet (Additional file 1: Fig. S1c) research center, respectively. The people living around Robe Bale commonly use *A.absinthium* (Additional file 1: Fig. S1b) against different ailments*. A.absinthium* was highly available and purchased from different markets in Ethiopia.

### Extraction and fractionation of Artemisia abyssinica

The leaves of *A.abyssinica* (Local name: *Ajoo in Afan Oromo*) (5 g) were air-dried and extracted with n-hexane/Ethyl acetate (8:2) for one day at room temperature which was then filtered and concentrated using a rotary evaporator to yield 2 g blackish oil crude extract. The crude extract (1 g) was then subjected to column chromatography using increased polarities of the n-hexane/ethyl acetate solvent system and yielded fractions 1–6 (Table 1).

**Table 1:** Fractionation of hexane/Ethyl acetate leaves for extract of *Artimisia abyssinica*

Fraction	Solvent systems	Amount (g)
1	n-hexane	0.4
2	n-hexane	0.3
3	n-hexane/EtOAc (9:1)	0.1
4	n-hexane/EtOAc (9:1)	0.025
5	n-hexane/EtOAc (8:2)	0.04
6	EtOAc	0.1

### Steam distillation of leaves of Artemisia abyssinica

A 100 g of leaves of the plant was ground and steam distilled for 3 h and the distillate was then poured into a Separatory funnel with 100 ml of chloroform. The organic phase was separated from the aqueous phase by using the Separatory funnel. The organic layer was dried using anhydrous Na_2_SO_4_ and filtered. The oil extracted had been concentrated using Rotary Evaporator and yielded yellowish oil (0.02 g).

### Extraction of leaves of and isolation of compounds from Artemisia annua

#### Isolation of artemisinin from A. annua leaves

Ground leaves of *A. annua* (200 g) were extracted with distilled water (150 ml), while shaking for 8 h and filtered using Whatman No. 1 filter paper. The filtrate was then added into a Separatory funnel and fractionated with 150 ml of n-hexane three times. The n-hexane extract of *A. annua* was then concentrated using a rotary evaporator and yielded a white crystal coded as AANH-1 (7 mg, 0.004%).

### Extraction of *A. abyssinica* and *A. absinthium* leaves

Leaves of *A. abyssinica* (50 g) and *A. absinthium* (50 g) were extracted with distilled water (50 ml) by shaking for 8 h. The extracts were then filtered using filter paper. The filtrate was then added into a Separatory funnel containing 50 ml of n-hexane each. The organic (n-hexane) layer was then fractionated from the aqueous layer three times. The n-hexane was then concentrated using a rotary evaporator and yielded white solid (30 mg, 0.06%) and (50 mg, 0.1%), respectively. These extracts were then compared with that of n-hexane of *A. annua* (Additional file 1: Fig. S1d) using TLC plate, solvent system n-hexane/EtOAc (7:3).

### Methanol extraction and isolation of compounds from leaves of *A. annua*

#### Methanol extraction of leaves of *A. annua*

Fresh leaves of *A. annua* were dried, powdered (Additional file 1: Fig. S1d) air-dried for a week. A 100 g of powdered leaves of *A. annua* were macerated using methanol three times and filtered. The extract was then evaporated using a rotary evaporator vacuum at a temperature of 40 °C. Distilled water (50 ml) was added to the methanol crude extract and partitioned using 50 ml n-hexane three times using a Separatory funnel. The aqueous layer was then further fractionated using 50 ml of ethyl acetate three times. Each extract was concentrated using a rotary evaporator at a temp of 40 °C and yielded n-hexane fraction (21 g, 21%) and ethyl acetate fractions (9 g, 9%).

### Purification of n-hexane fraction of leaves of *A. annua*

The most viscous *n*-hexane extract of the plant was fractionated by column chromatography using silica gel 60 as the stationary phase and a mixture of ethyl acetate-hexane with increasing polarity (gradient elution) as a mobile phase. Fractions 1–3 were obtained using n-hexane as an eluent and fractions 4–7 were collected using n-hexane–EtOAc (97:3) and fraction 8–10 were collected using n-hexane/EtOAc (9:1). Fractions 6 and 7 were found as amorphous compounds after concentrated with a rotary evaporator and showed single spots with the same Rf values with n-hexane–EtOAc (97:3). Then fraction 7 was submitted for NMR analysis. Fraction 6 was (Fig. 4c) stored at 4 °C and used for antimicrobial activity (antibacterial and fungal) and antimalarial study.

### Characterization of isolated compounds from *A. annua*

The ^1^H and ^13^C NMR were recorded in CDCl_3_ using the solvent peak as reference (chloroform: δH 7.26 and δC 77.10. Chemical shift values were reported in δ (ppm) units, the solvent signals as internal references. ^1^H, ^13^C, and DEPT spectra were obtained on a Bruker UltrashieldTM 400 spectrometer at 400 and 100 MHz, with TMS and solvents as internal standard and values were given in ppm relative to TMS internal standard.

### Tested microorganisms and antimicrobial activity assay

The standard strains used in this study were *Klebsiella pneumoniae* ATCC170^T^, *Escherichia coli* ATCC 25922^ T^, Hospital acquired *A. baumannii,.* S*taphylococcus aureus* ATCC 25923^ T^, *Salmonella enteritidis*ATCC13076^T^, *boydii* ATCC1233^T^, *Streptococcus pyogen ATCC* 19615^ T^ and *S. typhimurium* ATCC 13311^ T^ The strains were sub-cultured and incubated at 37 °C for 24 h using Nutrient Broth and was kept refrigerated on Nutrient agar slants for up to 2 weeks.

### Antibacterial activities test

Disk diffusion method and agar well diffusion were used to detect antimicrobial activities of crude extracts, fractions and essential oils leaves of the plants using a Muller Hinton agar media (Merck, Germany) (Beef extract, 2 g; Acid Hydrolysate of Casein, 17.5 g; starch, 1.5 g; Agar, 17 g).

### Disk diffusion method

Disk diffusion method of antibacterial activities was performed (Bauer et al. 1966). Briefly, Muller Hinton agar media was used as a culture medium. Concentrations of 1–2 × 10^8^ CFU/ml of bacterial inoculate were used. McFarland 0.5 was used as a standard control for bacterial inoculum. The media were poured onto 90 mm diameter petri plates until the thickness of the agar was 4 mm so that possible problems of diffusion of the tested products could be prevented. A 0.1 ml of each bacterial solution was inoculated and uniformly distributed onto the plates by means of sterile swabs. Plates were allowed to stand for 15 min. At the same time, 6 mm diameter disks were soaked/ impregnated with the crude extracts, fraction and essential oils of *Artemisia* spp using different concentrations. The impregnated disks were symmetrically placed onto the medium by using sterile tweezers. One of the disks was soaked with sterile distilled water and used as a negative control. The plates were incubated for 24 ± 2 h at 37ºC under anaerobic conditions. The results were evaluated by measuring the areas with no bacterial growth. These experiments were carried out in triplicates and control cultures were prepared for all the strains.

### Agar well diffusion

Agar well diffusion was also conducted following the methods of (Sz et al. 2016) with some modification. Briefly, about 15–20 ml of Mueller Hinton agar was poured onto glass plates of the same size and allowed to solidify. The agar surface of each plate was streaked by a sterile cotton swab with the reference bacterial strain. Agar plates were punched with a sterile cork borer of 6 mm diameter. A 100 μl of each sample of diluted crude extracts, fraction and essential oils of the plants were added into respective agar wells. The plates were allowed to standby for 30 min. Different Impregnated antibiotics disks or standard antibiotics were also used as positive control (Table 4). Three of the disks were soaked with sterile distilled water as a negative control. The plates were incubated at 37 °C for 48 h. The results were recorded by measuring the areas with no bacterial growth. These results were obtained after using the following formula: Inhibition value = Inhibition diameter in mm – Disk diameter (6 mm)/2. These experiments were carried out in triplicates and control cultures were prepared for these bacterial strains.

### Data analysis

One-way ANOVA was used to analyze the zone of inhibition due to some test extract obtained from *Artemisia* spp. Some data was also computed into tables and Excel. Post Hoc test was used to determine whether chemosuppression induced by each of the plant extracts was significantly different from the chemosuppression in the positive and negative control group.

## Result

### Thin layer chromatography analysis of artemisia species leaves extracts

About 10–20 μL sample of test extract compound was spotted onto TLC plate prepared using silica gel and allowed to run in the solvent system consisting of ethyl acetate and n-hexane (3:7) for 40 min at room temperature. TLC profile and their Rf values for *A. abyssinica*, *A. absinthium* that may contain artemisinin or other sesquiterpene lactones closer in structures with artemisinin were evaluated and required for further analysis.

### Isolation of artemisinin from *A. annua* leaves

**Fig. 1:**
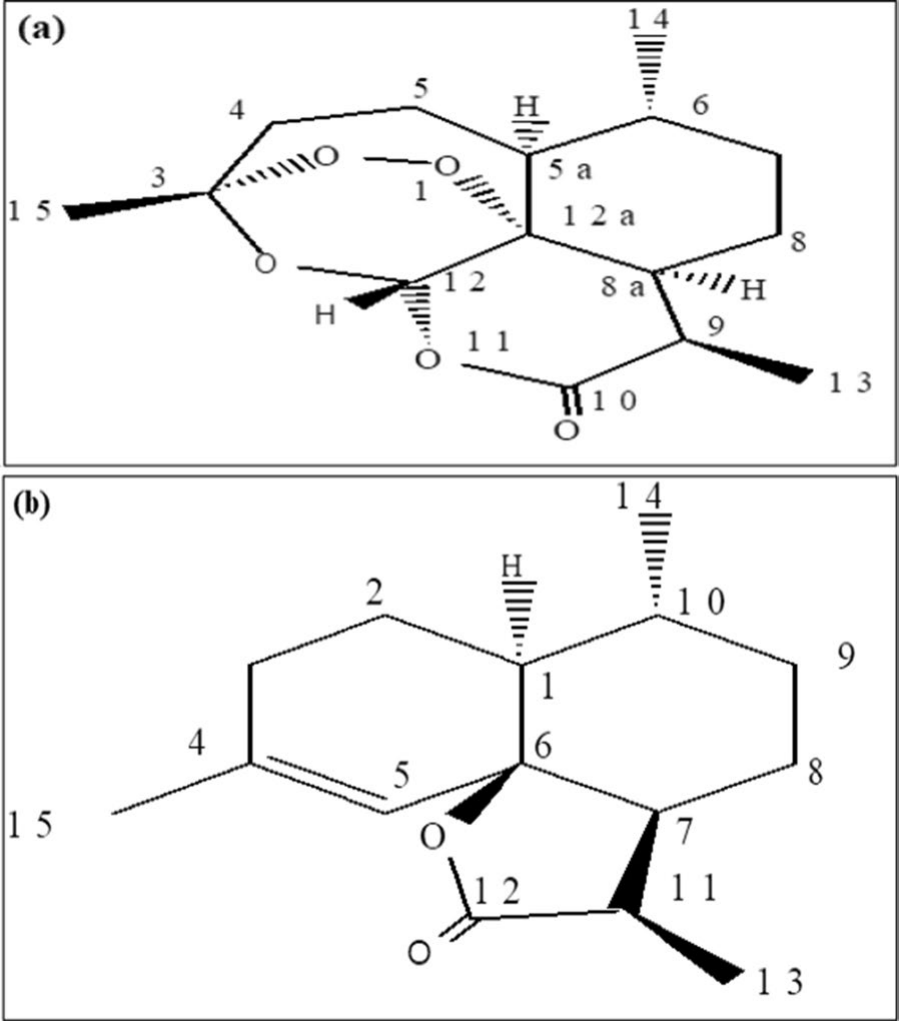
**a** Structure of artemisinin, **b** Structure of AANH-7

**Fig. 2:**
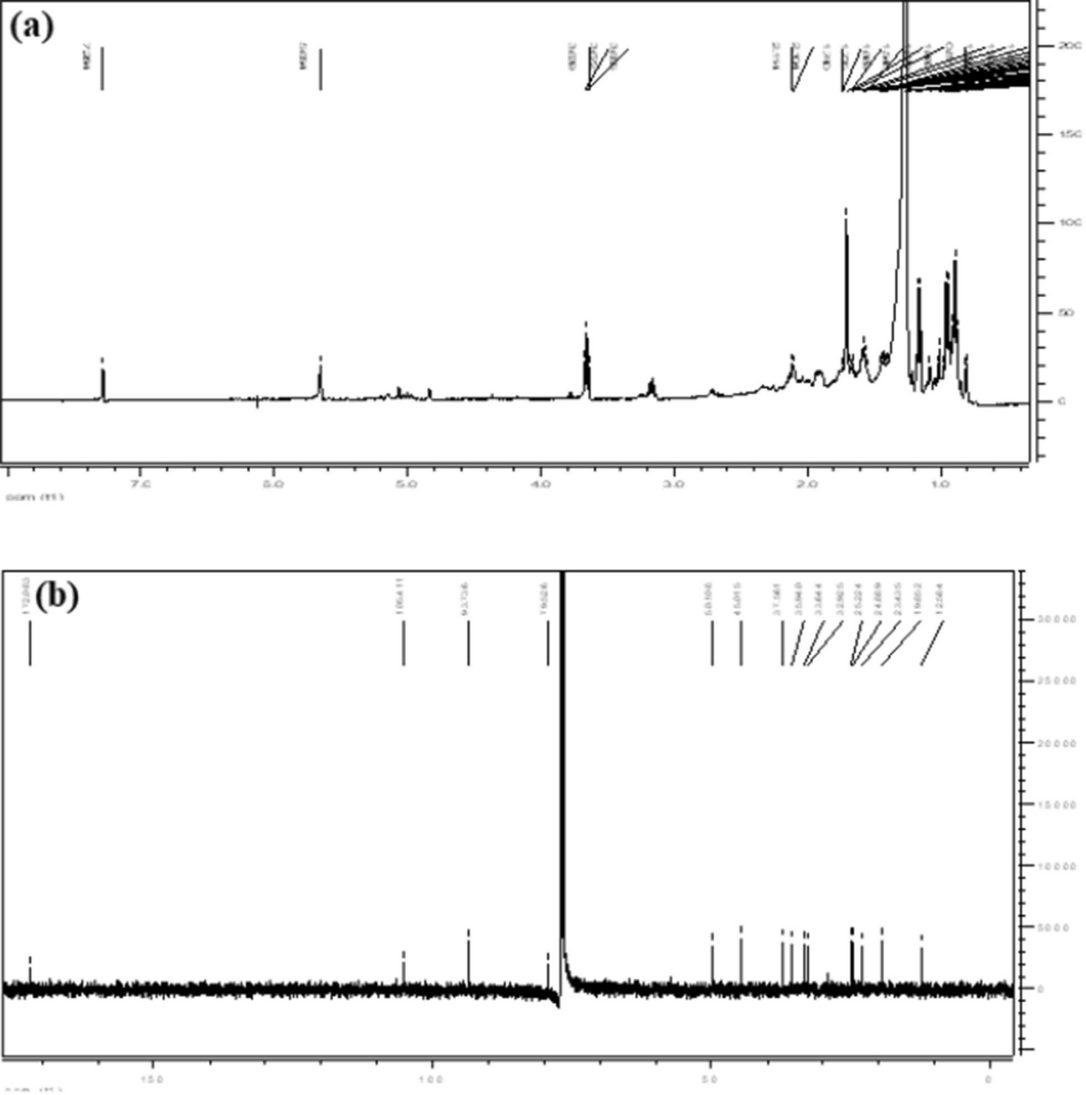
**a **^1^H NMR spectrum of artemisinin (AANH-1), **b** ^13^C NMR spectrum of artemisinin (AANH-1)

In the current study, Artemisinin compound (Fig. 1a) was extracted from the leaves part of *A. annua* using ethyl acetate extract, *n-*hexane, and petroleum ether. While extraction, a white crystal (mp 153–154 ºC) designated as AANH-1 (*Artermisa annua n-*Hexane fraction 1)was isolated from n-hexane extract of *A. annua* and analyzed by TLC and showed a single spot. The ^1^H-NMR of the compound showed a highly down field shifted signal 5.88 ppm which appeared as a singlet due to the presence of a proton attached to a carbon containing two oxygen (Table 2, Fig. 2a).

**Table 2:** ^1^H and ^13^C NMR spectral data of compounds AANH-1 and standard artemisinin

C/H	AANH-1*		Artemisinin**	
	δ_C_	δ_H_ (multi., J, Hz)	δ_C_	δ_H_ (multi., J, Hz)
3	105.4		106.7	
4	35.9	3.43 (m)	36.6	2.08 (ddd)
5	24.8	2.52 (m)	25.7	2.01 (m)
				1.47 (m)
5a	50.2	1.97 (m)	51.2	1.38 (m)
6	37.6	2.47 (m)	38.1	1.52 (m)
7	32.9	1.76 (m)	34.6	1.09 (m)
		2.47 (m)		1.77 (m)
8	24.8	1.48 (m)	24.0	1.17 (m)
8a	45.0	2.47 (m)	45.6	1.82 (m)
9	33.6	3.60 (m)	34.0	3.31 (dq)
10	172.1			
12	93.7	5.88 (s)	95.5	6.03 (dq)
12a	79.5		81.0	
13	12.6	1.22 (d, J = 7.34)	12.7	1.16 (d, J = 7.2)
14	19.8	1.02 (d, J = 5.36)	19.9	0.99 (d, J = 6.2)
15	25.2	1.60 (s)	25.2	1.38 (s)

* δ values are in ppm. ^1^H: 400 MHz; ^13^C:100 MHz; solvent and internal reference: CDCl_3_; multi: multiplicity ** (Margueritte et al. 2018) in CD_3_OD, 700 MHz). The AANH-1 was obtained from n-hexane extract of *A. annua* leaves

Additionally, ^1^H NMR spectrum indicated the presence of three methyl protons out of which two appeared as doublet and one signal singlet in the compound. The compound has 15 carbon signals (3 methyl signals, 4 methylene, 5 methine and 3 quaternary carbons) which indicated the compound is a sesquiterpene (Table 2, Fig. 2b).

### Characterization of compound AANH-7

Fraction 7 obtained from CC of n-hexane extract of *A. annua* as colorless oil. It’s TLC showed a single spot indicating the compound is pure. It was then analysed by ^1^H, ^13^C-NMR and DEPT which showed the compound has 15 carbons out of which 3 methyl carbons, 4 methylene carbons, 5 methine carbons and 3 quaternary carbons. ^13^C and DEPT spectra of the compound showed it has ester carbonyl carbon at 179.5 ppm and olefinic carbon at 142.3 and 121.8 ppm. The compound has also oxygenated quaternary carbon at 83.2 ppm (Table 3).

**Table 3:** ^1^H and ^13^C NMR spectral data of compounds AANH-7

C/H	AAN-7		Dibydro-epideoxyartannrrin B*	
	δ_C_	δ_H_ (multi., J, Hz)	δ_C_	δ_H_ (multi., J, Hz)
1	42.8	2.16 (m)	42.9	
2	23.7	1.93 (m)	23.8	
3	31.9	1.47 (m)	32.4	
4	142.3		142.5	
5	121.8	5.65 (s)	121.8	5.63 (s)
6	83.2		83.4	
7	39.6	2.19(m)	39.7	
8	21.0	2.13 (m)	21.0	
9	30.6	1.30 (m)	30.8	
10	32.4	1.23 (m)	32.7	
11	46.5	3.13 (m)	46.6	3.15 (dq. J1 = 7.0, J2 = 7 .2)
12	179.5		179.5	
13	9.4	1.24 (d, J = 7.1)	9.4	1.15 (d, J = 7.2)
14	19.8	0.93 (d, J = 6.6)	19.6	0.93 (d, J = 6.6)
15	23.4	1.71 (s)	23.5	1.75 (s)

δ values are in ppm. ^1^H: 400 MHz; ^13^C:100 MHz; solvent and internal reference: CDCl_3_; multi: multiplicity, *(Favero et al. 2014; Foglio et al. 2002). The AANH-7 was obtained from n-hexane extract of *A. annua* leaves

In this Study, sesquiterpene lactone dibydro-epideoxyartannrrin B. was identified and designed as AANH-7 (*Artermisa annua n-*Hexane fraction 7) (Fig. 1b). The ^1^H –NMR spectral data of the compound also shown olefinic proton at 5.65 ppm, methyl protons at 0.92 ppm (d, J = 6.6 Hz), 1.15 ppm (d, J = 7.1 Hz), and 1.75 ppm. The ^1^H-NMR, and ^13^C-NMR spectral data of this compound was in good agreement with those previously reported for dihydro-epideoxyarteannuin-B which was also reported from *A. annua* leaves (Favero et al. 2014; Foglio et al. 2002). The same authors further identified artemisinin, deoxyartemisinin and dihydro-epideoxyarteannuin-B from the same plant parts using GC–MS and ^1^H-NMR, ^13^C-NMR.

### Antibacterial activities

All test extracts of *A. abyssinica n-hexane extract, A. absinthium* ethyl acetate and *A. annua* n-hexane and petroleum ether showed varying degrees of inhibiting effect against bacterial strains such as *Escherichia coli* ATCC 25922^ T^, *Klebsiella pneumoniae* ATCC1053^T^, hospital-acquired *A. baumannii, Salmonella enteritidis* ATCC13076^T^, *boydii* ATCC1233^T^, and S*taphylococcus aureus* ATCC 25923^ T^*.* For all bacterial strains against test extract, different zones of inhibition were recorded (Table 4). During our study, the survey showed that the rural community used to treat the different infections using these traditional medicinal plants. These infections may be due to bacteria or fungi. Specially, *A. abyssinica* which is a native medicinal plant to Ethiopia in the area is a well-known traditional medicine for the healing of some infections.

**Table 4:** Antimicrobial sensitive test (Zone of inhibition in mm diameter) for *Artemisia annua* and *Artemisia absinthium* for petroleum ether and ethyl acetate extract

**Test strains**	**Zone of inhibition(mm) for Plant extract**			**Positive control (IZ = mm**)								
	**A.ah**	**A.ap**	**A.abe**	**TET**	**CIP**	**ERY**	**AMp**	**SXT**	**CHL**	**OX**	**DA**	**P**
*Hospital acquired A. baumannii*	34.00	15.00	35.00	21.00	16.00	no	no	–	–	–	–	no
*E. Coli ATCC 25,922* T	19.67	12	15.67	24.00	35.00	–	21.00	23.00	22.00	–	–	–
*Klebsiella pneumoniae* ATCC1053^T^	3.00	< 3.00	5.00	25.00	30.00	–	23.00	–	27.00	–	–	–
*Salmonella enteritidis* ATCC13076^T^	17.00	18.00	20.00	25.00	32.00	–	23.00	23.00	25.00	–	–	–
*Shigella boydii ATCC1233* ^T^	no	no	no	35.00	40.00	–	25.00	27.00	27.00	–	–	–
S.*aureus* ATCC 25923^ T^	12.67	17.5	20.33	–	–	24.00	–	20.00	20.00	25.00	25.00	40.00

A.ah- *Artemisia annua n-*hexane extract*,* A.ap—*Artemisia annua* petroleum ether extract and A. abe-* Artemisia absinthium* ethyl acetate extract

**Fig. 3:**
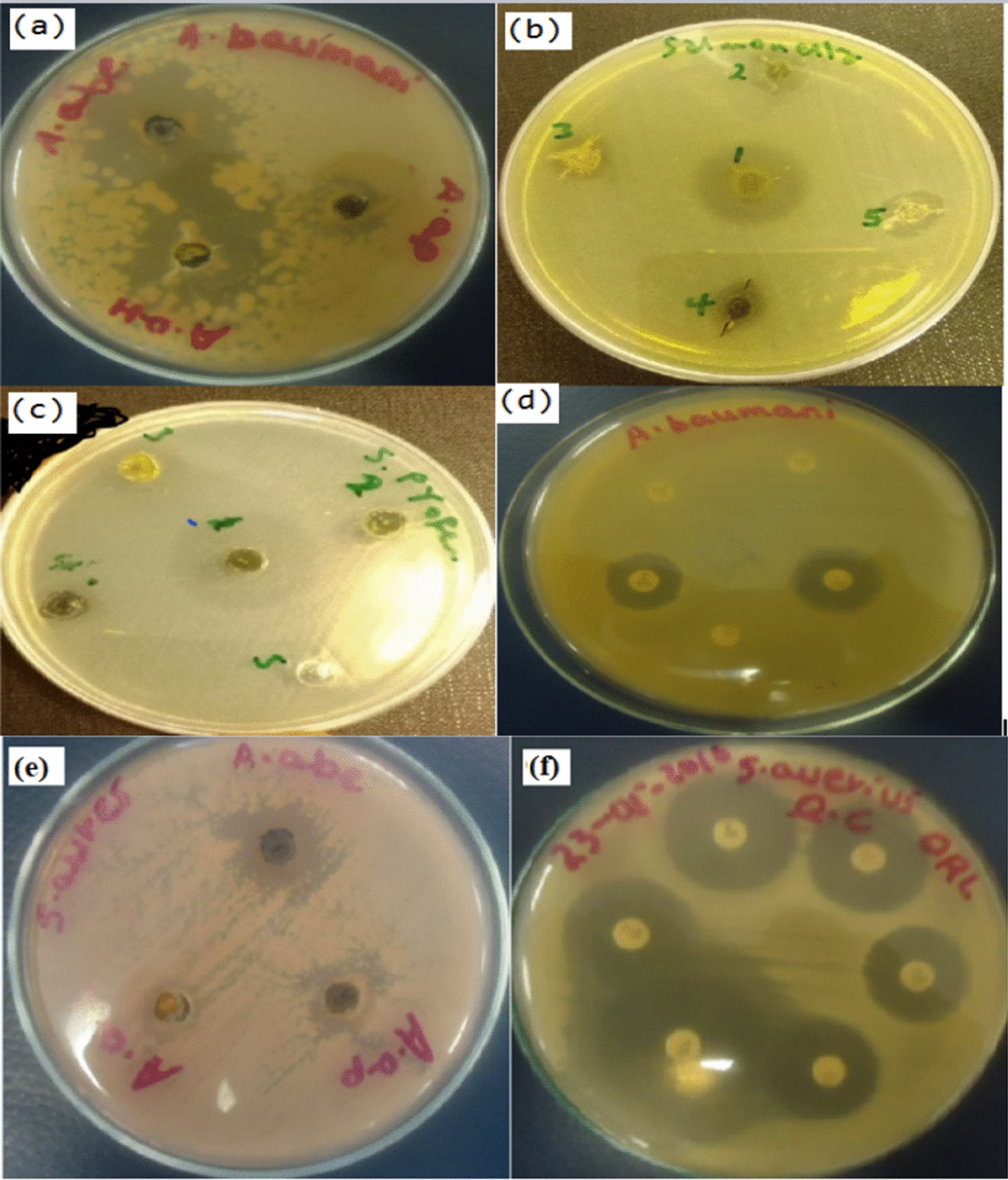
Zone of inhibition for Hospital acquired *A. baumannii* due to **a** plant extract of A.ah, A.ap and A.abe. **b** Zone of inhibition for *Salmonella* spp. **c** Maximum zone of inhibition for *S. pyogen ATCC* 19,615. **e** Zone of inhibition due to A. ab_e_, A.a_h_, and A.a_p_ against *Staphylococcus aureus*. **f** Zone of Inhibition due to some Standard impregnated disks

Our study showed that test extracts of *A. annua* and *A. absinthium* have oil. It was proved that these plants are used to produce a considerable amount of essential oil which might be employed to inhibit growth of some hospital-acquired bacterial pathogens such as hospital acquired *A. baumannii* (Fig. 3a). In this study, the crude extract of *A. absinthium* ethyl acetate extract (A.ab_e_) showed the maximum inhibiting effect (35 mm) (Table 4) and followed by *n-*hexane extract of *A.annua* (A.ah) against Hospital acquired *A. baumannii* in which zone of inhibition was 34 mm (Fig. 3a). The minimum zone of inhibition was recorded for (Table 4) test extract of A.ap.

The minimum inhibition zone (8 mm) was detected against *S. typhimurium* ATCC 13311^ T^ for both A.ac and EtOAc oils (8:2) of fractionated test extract (Table 6). The maximum zone of inhibition (25 mm, Table 5) for fractionated test extract of *A. abyssinica* (A.ach F4) was recorded against *S. pyogen ATCC* 19,615 (Fig. 3b). Significant variations (P = 0.887) were observed between all test extracts of these medicinal plants at 95% of confidence intervals. During this study, the minimum zones of inhibition were detected for the bacterial strains that were sensitive to the test extract of *Artemisia spp* in the disk diffusion assay. However, the maximum zone of inhibition was detected for the same medicinal plants in the agar well diffusion assay.

**Table 5:** Zone of inhibition against some strains of bacteria by test extract of *Artemisia abyssinica* at different concentration and fractionation

**Sample** **(A.ach)**	**Conc. (mg/ml)**	***S. aureus*** **ATCC 25923**^** T**^		***S. pyogen**** ATCC* 19,615		***E. coli ATCC 25,922* T**		***S. typhimurium***	
		**IZ (mm)**	**Sensitivity**	**IZ (mm)**	**Sensitivity**	**IZ (mm)**	**Sensitivity**	**IZ (mm)**	**Sensitivity**
A.ac_h_ F3	0.6	12.00	S	12.00	S	12.00	S	15.00	S
A.ac_h_ F4	1.30	20.00	S	25.00	S	15.00	S	17.00	S
A.ac_h_ F5	0.10	11.00	S	14.00	S	9.00	S	10.00	S
A.ac_h_ or EtOAc (8:2)	0.20	10.00	S	11.00	S	13.00	S	8.00	S
A.ac oil	0.08	10.00	S	12.00	S	20.00	S	8.00	S

Key: A.ac_h_—For n-hexane extract of *Artemisia abyssinica* (also known as “chikugn” or “Ajo” in Ethiopia). F indicate for fractionated test extract of *Artemisia abyssinica;* S is for sensitivity, IZ – is for Inhibition zone, R for Resistance. QC is for Ceftraxone and Ceftazidim

In the present study, our finding showed that Hospital acquired *A. baumannii* is highly sensitive for test extract of A.ah and A.abe with a range of zone of inhibition (15–35 mm) in diameter (Fig. 3c). The same bacterial species is highly sensitive for some standard impregnated commercial disks (Fig. 3d). In the current study, A.abe is especially more potent against the *staphylococcus aureus* (Fig. 3e&f). A significant variation was observed among A.abe & A.ah (P = 0.760), A.abe & A.ap (P = 0.625), A.abe & A.achF4 (P = 0.582), A.abe & A.achF3 (P = 0.582), and A.abe & AachF5 (P = 0.399), A.abe & Aach or EtOAc (P = 0.354) and A.abe & AaCh oil (P = 0.554).

**Fig. 4:**
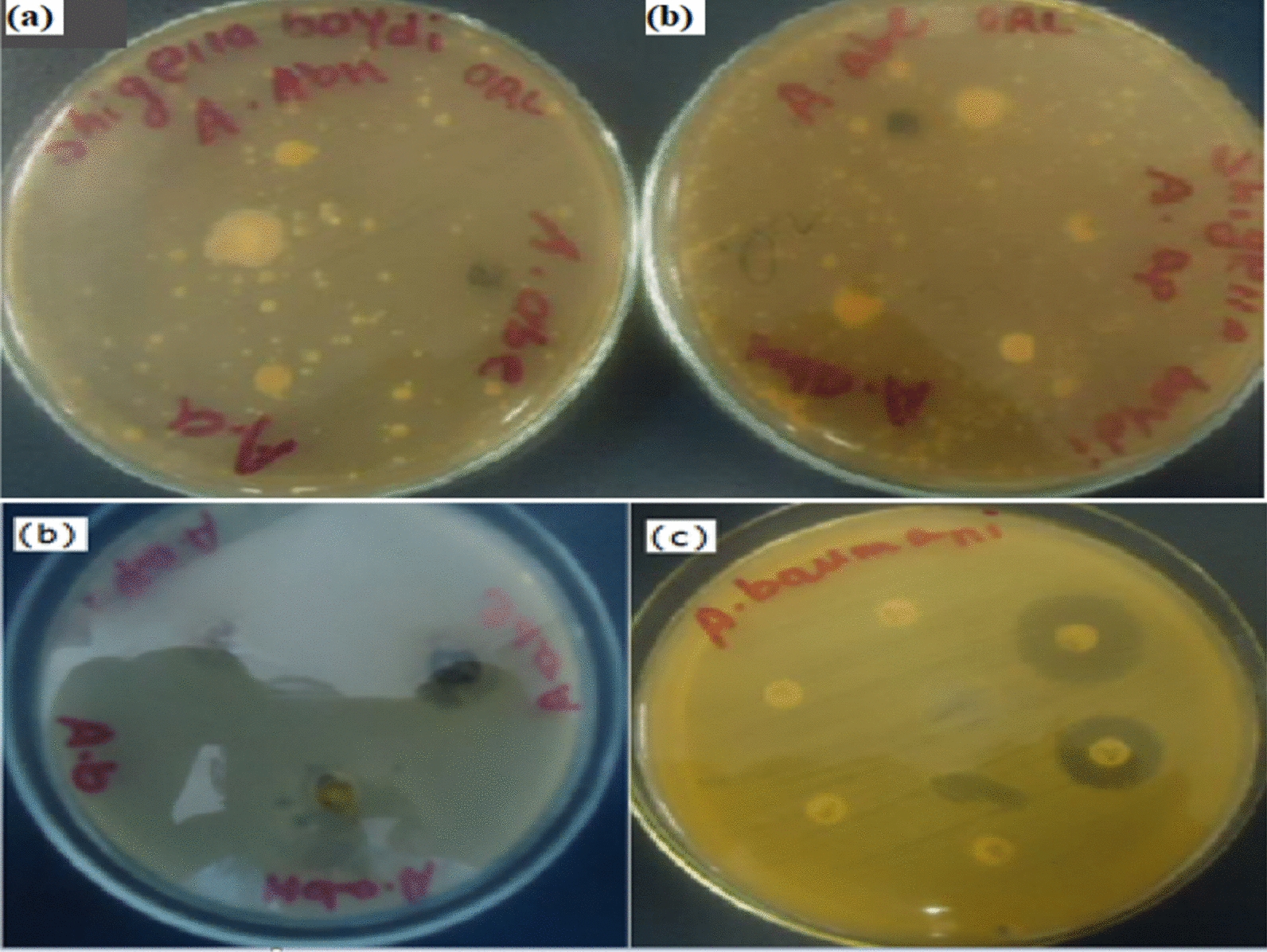
**a** No zone of inhibition was recorded for *Shigella boydii* ATCC 1233^ T^ **b** Synergistic effect of A.a_h_ and A. ab_e_ on Hospital acquired *A. baumannii (***c**). Zone of inhibition for some Standard impregnated disks. **d** Zone of inhibition against *Shigella boydii* by A.ap. extracts

In this study, the maximum zone of inhibition was 19.67 mm and the minimum inhibition zone was 12 mm against *Escherichia coli* ATCC 25,922 due to A.ah (Fig. 4a). For A.ach oil, the maximum zone of inhibition was recorded for *Escherichia coli* ATCC 25,922 and followed by A.achF4 which is 15 mm in diameter (Fig. 4b).

The test extract of A.ah, A.ap and A.abe were also used to inhibit the growth of *Klebsiella pneumoniae* ATCC1053^T^ which is a food borne bacterial pathogen. In the present study, the minimum zone of inhibition (< 3 mm or range between < 3 mm -5 mm) due these test extract is recorded for *Klebsiella pneumoniae* ATCC1053^T^. This bacterial spp may develop resistance against the test extract of A.ah A.ap and A.abe. In this study, both disk and agar well diffusion was conducted for *Shigella boydii* ATCC1233 ^T^ However, there was no zone of inhibition detected for *Shigella boydii* ATCC 1233 T (Fig. 4a). In this study, the test extract of A*. annua n-*hexane extract (A.ah) and *A. absinthium* ethyl acetate extract (A. abe) have synergistic effect on hospital acquired *A. baumannii*, a hospital acquired pathogenic bacterial spp (Fig. 4b).

In the present study, the distilled water was employed as negative control using agar well diffusion and disk diffusion against *Salmonella enteritidis* ATCC13076^T^. Standard impregnated disks were also employed as positive control. There is no zone of inhibition or growth for negative control (Additional file 1: Fig. S2a). But, a very clear zone of inhibition was detected for positive control due to some standard impregnated disks (Additional file 1: Fig. S2b). There was a best synergistic effect for Tetracycline, Ciprofloxacin and Chloramphenicol against *Salmonella enteritidis* ATCC13076^T^. At the same time, the test extract of *Artemisia* species such as A.ah, A.ap, A.ac and A.abe were used against the same bacterial species. A test extract of A.ap has inhibitory effect against *Salmonella enteritidis* ATCC13076^T^ with a very clear zone of inhibition (34 mm) (Additional file 1: Fig. S2c). For instance, traditionally the community uses *A. abysinthium* for treatment of gastric pain. As shown in Fig. 4d, no inhibition effect against *Shigella boydii* by A.ap. extracts.

## Discussion

Our Samples had been characterized by using NMR. The carbonyl signal at 172.1 ppm indicated the compound has an ester group. A highly downfield shifted methyne carbon signal at 105.4 ppm and a quaternary carbon signal at 93.7 ppm indicated the compound is highly oxygenated. The ^13^C-, DEPT, ^1^H -NMR spectral data of the compound was identified by comparison of its ^1^H, ^13^C NMR spectral data with those reported for artemisinin (Cafferata et al. 2009; Margueritte et al. 2018). There were different methods of extraction and purification for artemisinin. Dahnum et al. (2012) isolated artemisinin using extraction of *A. annua* with methanol while stirring, portion of the methanol extract with n-hexane followed by column chromatography. It has been confirmed that artemisinin can be purified from *A. annua L.* using (Appalasamy et al. 2014) Pressurized Hot Water, Soxhlet extraction and maceration with method at 60 °C followed by HPLC **(**Hao et al. 2002; Sixt and Strube 2017). Tzeng et al. (2007) obtained pure artemisinin by normal column chromatography of ethanol modified SC-CO_2_ extractions of whole parts of *A. annua*. The percentage yield of artemisinin (0.004%) we obtained in this study is lower than those reported by Dahnum et al. (2012) (0.016%), and (ElSohly et al. 2004) (0.12%). Our methods of purification are fastest with less solvent and purification processes and thus economical for production of the antimalarial drug from *A. annua* leaves when compared with previously reported methods. In the current study, the artemisinin compound was obtained by using a separatory funnel. However, Hao with his co-authors **(**Hao et al. 2002**)** obtained artemisinin compound from extract of *A*. *annua L* by using Microwave-assisted extraction and Soxhlet method. The same authors employed chloroform, cyclohexane, ethanol, *n*-hexane petroleum ether, petroleum ether and trichloromethane for artemisinin extraction.

The ^1^H –NMR spectral data of the compound also indicated the presence of protons on olefinic proton at 5.65 ppm, methyl protons at 0.92 ppm (d, J = 6.6 Hz), 1.15 ppm (d, J = 7.1 Hz), and 1.75 ppm (s) appeared as a. Based on the above spectral data the structure of the AANH-7 was identified as a sesquiterpene lactone dibydro-epideoxyartannrrin B. The compound was previously reported from the stem and leaves *A. annua* (Brown 2004). The ^1^H and ^13^C NMR data of AANH-7 was in good agreement with that reported compound by Bilia et al. (2008) by using GC–MS analysis. It has been reported that (Foglio et al. 2002) dibydro-epideoxyartannrrin B when administered orally (100 mg/kg) on the indomethacin ulcer model inhibited the ulcerative lesion index with ED_50_ values of 55.6 mg/kg indicating the compound have a relationship with an increase prostaglandin synthesis.

In the present study, artemisinin was obtained from the test extract of A.ap and A.ah.. All test extracts contain certain essential oil. These test extracts may also contain some other aromatic compounds. These *Artemisia* species extracts were also shown to have inhibition effects. These inhibition effects could be due to the presence of artemisinin, sesquiterpene, and other aromatic compounds. In line with this study, it has been reported that (Hanscheid and Hardisty 2018) artemisinin that extracted from *A. annua* have shown antimalarial therapy, The same authors stated that microbial cells might be developed resistance against artemisinin compounds if it will be added to the list of choice drugs. Similarly in our study, no inhibition was observed for *Shigella boydii ATCC1233*^T^ suggesting that this pathogenic bacterial strain may develop resistance toward *A. annua* extracts that are predicted to be among artemisinin compounds. It was found that the essential oils derived from *A. absinthium* were extracted using microwave assisted process, distillation in water and direct steam distillation methods. These extracts of *Artemisia* species were shown for their relative toxicity against ascaricides and spider mite, *Tetranychus urticae* (Chiasson et al. 2001). Studies were shown that a sesquiterpene (C15H24) compound that was derived from *A. absinthium* by using present direct steam distillation (DSD) contained essential oil after the Chromatographic analysis had been performed. These oils have shown lethal effects against adult *Tetranychus urticae* (Chiasson et al. 2001).

Certain total phenolic contents have been detected for *A. absinthium* leaves extracts. This extraction was determined by using the Folin-Ciocalteu (FC) method. These phenolic compounds were included such as benzoic acid, Catechins, flavonols, hydroxycinnamic acids, hydroxybenzoic acids, and Gallic acid (Carvalho et al. 2011). The same authors predicted that these phenolic compounds were employed as antioxidants. A reversed-phase high-performance liquid chromatography method (RP-HPLC) coupled with diode-array detection (DAD) and electrospray ionization mass spectrometry (ESI/MS) analysis have shown that certain phenolic compound such as flavonoids (O- and C-glycosylated) and hydroxycinnamic acids derivatives were detected for *A. argentea*. These phenolic compounds are extracted by using methanol and measured by the Folin-Ciocalteu method (Gouveia-Figueira and Castilho 2011)*.* These authors added that these phenolic compounds have antioxidant capacity. Similar results were observed with other species of *Artemisia*. Carvalho et al. (2011) isolated both phenolic content and flavonoid compounds from *Artemisia* species*.* These compounds are determined by using Folin–Ciocalteau’s reagent and methanolic AlCl_3_·6H_2_O, respectively (Carvalho et al. 2011). Similarly, study has shown that the phenolic compounds have been separated by using HPLC method when the *A. argentea* extracts had been performed by using alcoholic methods of extraction (Gouveia-Figueira and Castilho 2011). For instance, the alcoholic extract of *A.argentea* was shown to contain a 152.8 mg 100 g DW-1 total phenolic content and a 109.20 mg 100 g DW-1 flavonoid content i for a given plant material (Carvalho et al. 2011). The phenolic compounds are the dominant antioxidants that show scavenging efficiency due to the presence of free radical compounds which is a reactive oxygen species. These free radicals are commonly reported for diversity of plant species (Prior et al. 2000). For instance, extract of *A. argentea* shown high radical scavenging capacity with totally free of harmful components such as *Artemisia* ketone and thujone (Gouveia-Figueira and Castilho 2011).

Previous studies have shown that artemisinin can be obtained from *A. annua* using microwave-assisted methods of extraction (Hao et al. 2002). The same authors stated that the artemisinin employed to act as antimalarial actvities. Other natural products may be existed within these *Artemisia* species in addition to artemisinin compounds that detected for specifically *A. annua.* The presence of natural antioxidants such as alkaloid, flavonoids, phenolic compounds, and terpenes in the aerial parts of A. *abysssinica* party elaborates the observed effects of plant extract (Taramelli et al. 1999) which is a similar finding to our suggestion. Other compounds with tR = 5.0 min had been identified as 5-O-caffeoylquinic acid *A. argentea* (Gouveia-Figueira and Castilho 2011). The same authors reported catechins, ferulic and caffeic acid from *A. argentea* and other six related species.

It has been reported that certain chemical components of the essential oil (91–97.1%) were predicted for *A. annua.* These essential oil components were found to be varying from 0.3–0.7% during the growth period. The major compositions were identified as borneol (7.5%), camphor (22.8–42.6%), β-caryophyllene (2–9.2%), 1,8-cineole (3.7–8.4%), (*E*)-β-farnese (1.3–8.5%), and germacrene D (0.5–7.3%). Meanwhile, other chemical components such as 1-*epi*-cubenol (0.7–5.2%). linalool (0.1–11.9%), β-pinene (6.5%), sabinene (8.2%), and β-thujone (9.8%) were identified from an extract of *A. annua*. These chemical compositions were characterized by using two-dimensional GC time-of-flight mass spectrometry (MS) (Abad et al. 2012a, b; Ma et al. 2007; Padalia et al. 2011)*.* After GC–MS and GC analysis had been performed, major components of essential oils such as myrcene, trans-thujone and trans-sabinyl acetate with 10.8, 10.1, and 26.4%. percentage yields were obtained from *A absinthium,* respectively (Lopes-Lutz et al. 2008). These essential oils showed moderate inhibitory effects against certain microbial cells such as *Candida albicans* and *Staphylococcus aureus.* However, the same essential oils were shown weak activities against *Escherichia coli*, *Proteus vulgaris,* and *Salmonella typhimurium* (Abad et al. 2012a, b; Ma et al. 2007; Padalia et al. 2011)*.* These discrepancies in terms of degree of inhibition effect could be due to variation of chemical composition found in these essential oils.

Lopes-Lutz et al. (2008) investigated the chemical composition and antimicrobial activity of essential oil isolated from aerial parts of *A. absinthium*, *A. biennis*, *A. cana*, *A. dracunculus*, *A. frigida*, *A. longifolia* Nutt. and *A. ludoviciana* using GC/MS. The same authors confirmed that Artemisia oils had inhibitory effects on the growth of some pathogenic bacteria and fungi. These pathogens are *Escherichia coli*, *Aspergillus niger, Candida albicans*, *Cryptococcus neoformans, Fonsecaea pedrosol. Microsporum canis*, *Microsporum gypseum*, *Staphylococcus aureus, Staphylococcus epidermidis,* and *Trichophyton rubrum* (Lopes-Lutz et al. 2008)*.*Certain fungi disease may be targeted due to some natural products these available within part of *Artemisia* species. In agreement with this prediction, the dried leaves of *A. annua* (Jiao et al. 2018) have been shown to be effective against avian coccidiosis which is a fungi disease. These natural products may be existed within leaves or root parts of *Artemisia* species. For instance, in the current study the maximum inhibition zone were detected for ethyl acetate extract using dried and powdered *A. annua* leaves part, a similar finding to (Jiao et al. 2018). The same authors stated that *Artemisinin* and *A. annua* leaves alleviate *Eimeria tenella* infection by facilitating apoptosis of host cells and suppressing inflammatory response.

Some unidentified inhibitory compounds may be used to damage certain structures of bacterial cells. Test extract of *A. absinthium* ethyl acetate extract (A.abe) was more effective against these selected bacterial strains and their zone of inhibition ranged from 5 to 35 mm. It was found that the whole part of *A. absinthium* ethyl acetate and chloroform extracts used to inhibit test microorganisms such as *Staphylococcus aureus* ATCC 25923^ T^, *Pseudomonas fluorescens, Bacillus brevis* FMC and *Bacillus megaterium* DSM with 8–16 mm/20 ml inhibition zone (Erdogrul 2002) which is strongly in agreement with our current results.

The essential oil of one of *A. absinthium*, also showed antibacterial activity against commonly known pathogens like *Escherichia coli*, *Salmonella enteritidis*, *Pseudomonas aeruginosa*, *Klebsiella pneumoniae* and *Staphylococcus aureus* (Blagojević et al. 2006). In agreement with this finding, an ethyl acetate oil extract of *A. absinthium* (A.ab_e_) tends to show the maximum inhibiting effect (35 mm) against Hospital acquired *A. baumannii*. This extract might have contained inhibiting inhibitory compound that able to target this pathogenic strain. In line with this study, it was stated that the GC/MS used to show the chemical composition and its antimicrobial activity of essential oil extracted from aerial parts of *A. absinthium*, *A. cana*, *A. biennis*, *A. dracunculus*, *A. frigida*, *A. longifolia* Nutt, and *A. ludoviciana* of wild sages from western Canada (Lopes-Lutz et al. 2008). The same authors briefly reported that *Artemisia* oils able to inhibit growth of pathogenic bacteria such as *Escherichia coli*, *Staphylococcus aureus* and *Staphylococcus epidermidis*. Previous reports indicated that the different species of *Artemisia* have a wide array of biological activities including antimalarial, cytotoxic, antihepatotoxic, antibacterial, antifungal, and antioxidant activity (Bora and Sharma 2011).

Fungi species such as *Candida albicans* and *Cryptococcus neoformans* were similarly inhibited by extract oil of *Artemisia*). *Aspergillus niger, Fonsecaea pedrosol Microsporum canis*, *Microsporum gypseum*, and *Trichophyton rubrum* are well known dermatophytes that were inhibited by *Artemisia* oils (Lopes-Lutz et al. 2008). It was found that the *A. absinthium* essential oil contained component such as β-thujone (10.1%), myrcene (10.8%), and *trans*-sabinyl acetate (26.4%). *A. biennis* extract oil contains the acetylenes (*Z*), (*E*)-en-yn-dicycloethers (11%), cis-β-ocimene (34.7%), and *trans*-β-farnesene (40%). *A. dracunculus* oil contained methyl chavicol which is predominant phenylpropanoids and methyleugeno (16.2%) (Abad et al. 2012b).

It has been reported that water, methanol, ethanol, or acetone extracts of artemisinin which are derived from *A.annua* L. have the ability for anti-inflammatory, antioxidant, and antimicrobial. The acetone extract is a well-known candidates of inhibitory effect on lipopolysaccharide-induced nitric oxide (NO), prostaglandin E2 (PGE2), and proinflammatory cytokine (IL-1β, IL-6, and IL-10) production. However, the ethanol extract have the best antioxidant activity due to its highest free radical scavenging activity (91.0 ± 3.2%), similar to α -tocopherol (99.9%) (Kim et al. 2015).

The Methanol extract of the *A.vulgaris* showed the highest antioxidant and antibacterial properties when compared to the essential oil of the same plants. It has also been stated that the artemisinin compound has the ability of inhibitory effect against *actinomycete mcomitans*, *Aggregatibacter,*, *Fusobacterium nucleatum* subsp. *animalis*, *Fusobacterium nucleatum* subsp. *polymorphum*, *periodontopathic* and *Prevotella intermedia* microorganisms. For instance, methanol extract used to inhibit *F. nucleatum* subsp*. Polymorphum* and *Prevotella intermedia* which is similar to the current finding (Kim et al. 2015). Johnson et al. (2013) further stated that the methanol solutions of the extracts were found to have broad-spectrum activity against all the microorganisms tested. In the present study, the maximum zone of inhibition for S*taphylococcus aureus* ATCC 25,923 (Fig. 3) was 20.33 mm and 20 mm in diameter (Table 4, 6) due to A.abe and A.ach F4, respectively which is more potent than the findings of Johnson et al. (2013).

**Table 6:** Zone of inhibition against some strains of bacteria by test extract of *Artemisia abyssinica* at different concentrations

Test extract	Conc (mg/ml)	*A. baumannii*		*E. coli* *ATCC* 25,922 T		*K. pneumonia* ATCC1053^T^		*S. enteritidis *ATCC13076^T^		*Shigella boydii* ATCC1233 ^T^		*S. aureus* ATCC 25923^ T^	
		IV/IZ mm	S	IV/IZ mm	S	IV/IZ (mm)	S	IV/IZ (mm)	S	IV/IZ (mm)	S	IV/IZ (mm)	S
A.a_h_	0.11	14/34	S	6.84/19.67	S	3.00	M	5.5/17	M	no	-	3.34/12.67	M
A.a_p_	0.11	4.5/15	M	3/12	M	< 3.00	M	6/18	M	no	-	5.75/17.5	M
A.ab_e_	0.03	14.5/35	S	4.84/15.67	M	5.00	M	7/20	M	no	-	7.17/20.33	S

Key: A.ach—For test extract of *Artemisia abyssinica* (also known as “*chikugn*” or “*Ajo*” in Ethiopia). F – is for fractionated test extract of *Artemisia abyssinica,* S is for sensitivity, IZ- for inhibition zone, IV – is for Inhibition Value. S = sensitive, R = Resistance Antibiotics used for QC: Ceftraxone and Ceftazidime, M- for moderately shown inhibition zone

The interaction in the oil constituents resulted in a synergy effect on microbial spp. except for *Salmonella typhi* and *Escherichia coli* ATCC 25,922 with a zone diameter of 6 mm each (Johnson et al. 2013). The same author further stated that a minimum zone diameter (6 mm) was observed for *Salmonella typhi and Escherichia coli* strains. However, the maximum zone of inhibition were recorded for *Candida albicans* and *Candida albicans* ATCC 90,028 (30 mm) strains when Tangerine oil extract had been used (Johnson et al. 2013).

In line with this study, (Patil et al. 2011) extracted and obtained physiologically active composition in pure or mixture form from some *Artemisia* sp. such as *A. dracunculus*, *A. herba-alba*, *A. judaica*, *A. vulgaris*, *A. abysinica*, *A. absynthicum*, *A. afra, A. cannariensis*, *A. pallens*, *A. annua*, *A. abrotanum*, *A. ludoviciana*, and *A. capillaris* or *A. scoparia* (Patil et al. 2011)*.* Moreover, the same author stated these plants used to prevent or treat (pre) diabetes and associated accompanying diseases or secondary diseases. The best zone of inhibition for essential oil of *Boswellia papyrifera* for bacteria was obtained for *Salmonella enterica* CIP 105,150 (40 mm), *Bacillus cereus* LMG 13,569 (39 mm), *Enterococcus faecalis* CIP 103,907 (39 mm), *Shigella dysenteria* CIP 5451 (31 mm), *Staphylococcus camorum* LMG 13,567 (30 mm). The other strains had sensitivities between 15–28 mm. The best zone of inhibition of methanol extract of *Boswellia papyrifera* were obtained for *Enterococcus faecalis* CIP 103,907 (30 mm), *Bacillus cereus* LMG 13,569 (27 mm). The other strains had sensitivities between 6–24 mm (Abdoul-latif et al. 2012).

The bacteria are resistant to testing an extract of all *Artemisia* spp. This might be due to the inappropriate concentration of test extract. Or the test extract might be inefficient to inhibit this bacterial strain. Abdoul-latif et al. (2012) is also stated that *Proteus mirabilis* CIP 104,588 is resistant to the methanol extracts of *Boswellia sacra* and *Boswellia papyrifera* while *Shigella dysenteria* CIP 5451 is resistant to the methanol extract of *Boswellia sacra* only. Essential oils of *Boswellia sacra* and *Boswellia papyrifera* present an antimicrobial activity stronger than the ticarcycline for *Enterococcus faecalis* CIP 103907^ T^, *Escherichia coli* CIP 105182^ T^, *Shigella dysenteria* CIP 5451^ T^, *Staphylococcus camorum* LMG 13567^ T^, *Pseudomonas aeruginosa and Proteus mirabilis* CIP 104588^ T^ (only for essential oil of *Boswellia sacra*). The methanol extracts of *Boswellia sacra* and *Boswellia papyrifera* have an antimicrobial activity weaker than the ticarcycline except for *Escherichia coli* CIP 105,182 (Abdoul-latif et al. 2012).

In Afro-Asian countries, many species of *Artemisia* such as *A. abyssinica* are used in folk medicine as anthelmintics, antispasmodics, antirheumatics and antibacterial agents due to the presence of the presence of certain natural products such as alkaloids, anthraquinones, flavonoids, sterols, tannins, and volatile oils (Adam et al. 2000). Certain essential oils are also reported from *A. asiatica* Nakai*.* These essential oils have shown antibacterial and antifungal effects. They include such as 1, 8-cineole, selin-11-en-4alpha-ol and monoterpene alcohols fraction. These essential oils have been found to be effective against *Bacillus subtilis, Aspergillus fumigatus, Candida albicans, Escherichia coli, Rhodotorula rubra, Pseudomonas aeruginosa* and *Staphylococcus aureus.* The monoterpene alcohols have specifically shown inhibiting effects against certain bacterial cells (Kalemba et al. 2002). In our study, it was confirmed that *A. annua* have shown antimicrobial properties. It could be due to the presence of secondary metabolites, a work similar to Appalasamy et al. (2014). In agreement with our finding, (Appalasamy et al. 2014) extracted the main inhibitory compound which is artemisinin from *A. annua* L. that collected from Malaysia. Due to the tropical hot climate, *A. annua* could not be planted for production of artemisinin, the main inhibitory compound. The same authors found out that the *A. annua* L. leaves extract is able to inhibit certain gram-negative and Gram-positive and Gram-negative bacteria. However, the leaf extract of *A*. annua L is unable to inhibit *Candida albicans* (Appalasamy et al. 2014). It was also reported that *A. annua* employed to act as therapeutic agent for the treatment of cancer (Chen et al. 2014), tuberculosis and diabetes (Li et al. 2017; Zheng et al. 2017)..However, in our study, inhibition activities were detected for *A. annua* petroleum ether extract*, A. absinthium* ethyl acetate extract and *A. abyssinica* were detected against certain medically important bacterial pathogens such as *E. Coli ATCC 25,922* T*, Hospital acquired A. baumannii, Salmonella enteritidis* ATCC13076^T^ and *S. aureus* ATCC 25923^ T^. This could be due to the presence of Artemisinin and other sesquiterpene compounds. These compounds may target certain bacterial structures such as cell wall, cell membrane, genetic material or ribosome that are used for protein synthesis.

In the current study, this *A. absinthium*, a traditionally known as *Ariti* has been shown inhibitory effects against certain pathogenic bacterial cells such as *E. Coli ATCC 25,922* T*, Hospital-acquired A. baumannii, Klebsiella pneumoniae* ATCC1053^T^, *Salmonella enteritidis* ATCC13076^T^ and *S. aureus* ATCC 25923^ T^. Mostly, in Ethiopia these *Artemisia* species are employed for ritual during solemn ceremonies.

The *A. abyssinica* has shown inhibitory effects against certain bacterial pathogens. These *Artemisia* spp are traditionally referred to as *Ajo.* It is highly grown some highland environments. These plants are commonly employed for house cleaning rather than as traditional medicinal plants. It has repellent and pungent odors. The same *Artemisia* spp. is traditionally known as *Ather* in Saudi Arabia (Adam et al. 2000), where these plants are abundantly grown. The same authors classified *A. abyssinica* under the family of Asteraceae. Furthermore, it was stated that *A. abyssinica* have shown certain effects on the growth, haematological (treatment of the blood) and organ pathology in rats at a low concentration (Adam et al. 2000). The test extract of *Artemisia* species such as A.ah, A.ap A.ac and A.abe were used against certain bacterial species. A test extract of A.ap has inhibitory effect against *Salmonella enteritidis* with a very clear zone of inhibition (34 mm) (Additional file 1: Fig. S2c). It was found that leaves and aerial parts of extract for *Artemisia* species such as *A.absinthium*, *A. abyssinica*, A*. afra,* and *A. annua* have been found to be effective against *Trypanosoma brucei brucei* with in Ethiopia (Nibret and Wink 2010).

## Supplementary Information

**Additional file 1.** **Fig. S1:** (a) Bale Robe (Enidato Gasera)- is area where Artemisia absinthium (Arity) was collected. (b) Artemisia absinthium (c) Wondogenet Agricultural research center, area where Exotic A. annua, Artemisia absinthium and other Ethiopian endogenous medicinal plants were collected. (d) Dried and ground A. annua. **Fig. S2:** (a) Zone of inhibition was detected for negative control. (b) Considerable amount of zone of inhibition was detected for test extract of Artemisia species especially with very clear zone of inhibition due to A.ap. (c) Acceptable zone of inhibitions were also detected for some impregnated antibiotics.

## Data Availability

All discussed data have been included into this manuscript.
