# Supplementary material for: Phytochemical analysis and anti-microbial activities of Artemisia spp. and rapid isolation methods of artemisinin
Source: AMB Express. 2022 Feb 12;12:17. doi: 10.1186/s13568-022-01346-5 (PMC8840944; doi:10.1186/s13568-022-01346-5)
Supplement: Supplementary file 1 — Additional file 1. Fig. S1: (a) Bale Robe (Enidato Gasera)- is area where Artemisia absinthium (Arity) was collected. (b) Artemisia absinthium (c) Wondogenet Agricultural research center, area where Exotic A. annua, Artemisia absinthium and other Ethiopian endogenous medicinal plants were collected. (d) Dried and ground A. annua. Fig. S2: (a) Zone of inhibition was detected for negative control. (b) Considerable amount of zone of inhibition was detected for test extract of Artemisia species especially with very clear zone of inhibition due to A.ap. (c) Acceptable zone of inhibitions were also detected for some impregnated antibiotics. [file 13568_2022_1346_MOESM1_ESM.pdf]

Phytochemical analysis and Anti-microbial activities of *Artemisia* spp. and Rapid isolation methods of artemisinin

Seid Mohammed[1]*, [2]Aman Dekabo and [1]Tilahun Hailu

[1]Adama Science and Technology University, Ethiopia

[2]Department of Applied Chemistry

E-mail: seid.mohammed@astu.edu.et

*Author for correspondence: Seid Mohammed Ebu

[1]Applied Biology Department

School of Applied Natural Science

Mobile (Current and Home): +251-912-223580*

Address (current and Home): Adama Science and Technology University, Oromia, Ethiopia*

Running Title: Full research article

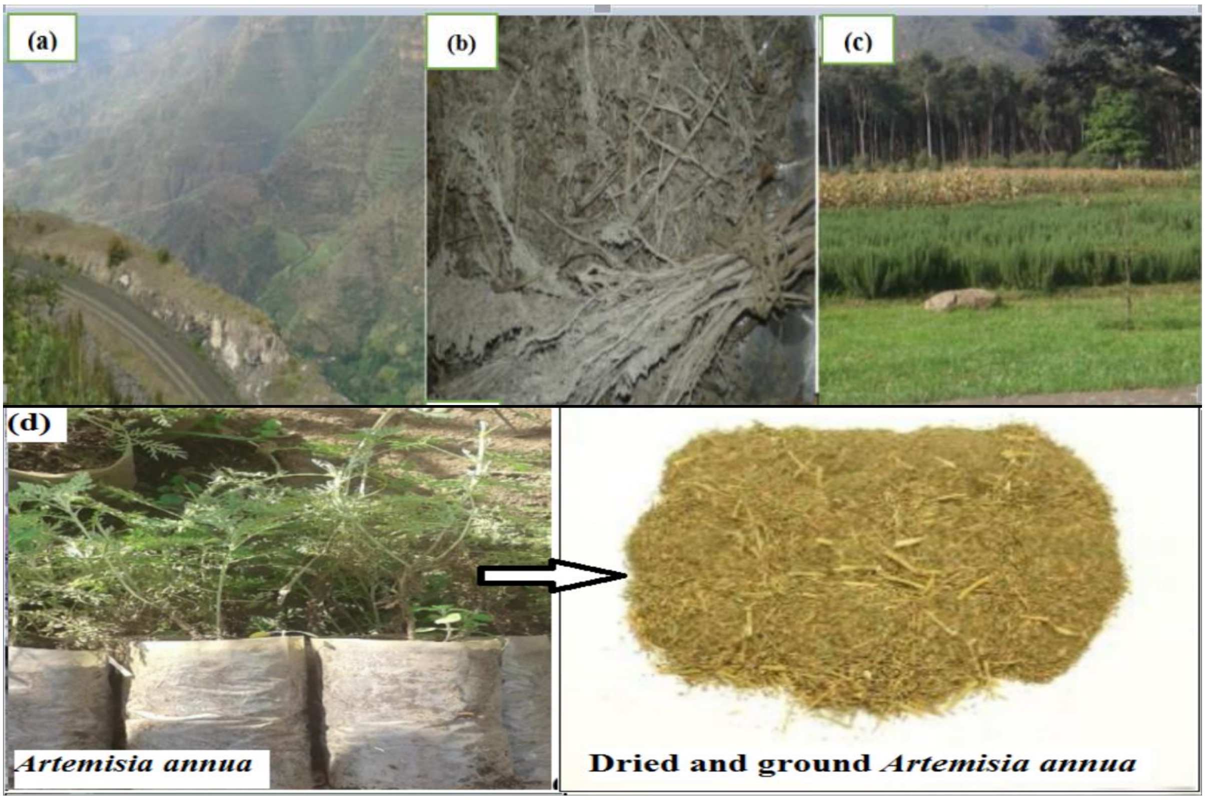

Fig. S1: (a) Bale Robe (*Enidato Gasera*)- is area where *Artemisia absinthium (Arity*) was collected. (b) *Artemisia absinthium* (c) Wondogenet Agricultural research center, area where Exotic *A. annua*, *Artemisia absinthium* and other Ethiopian endogenous medicinal plants were collected. (d) Dried and ground *A. annua.*

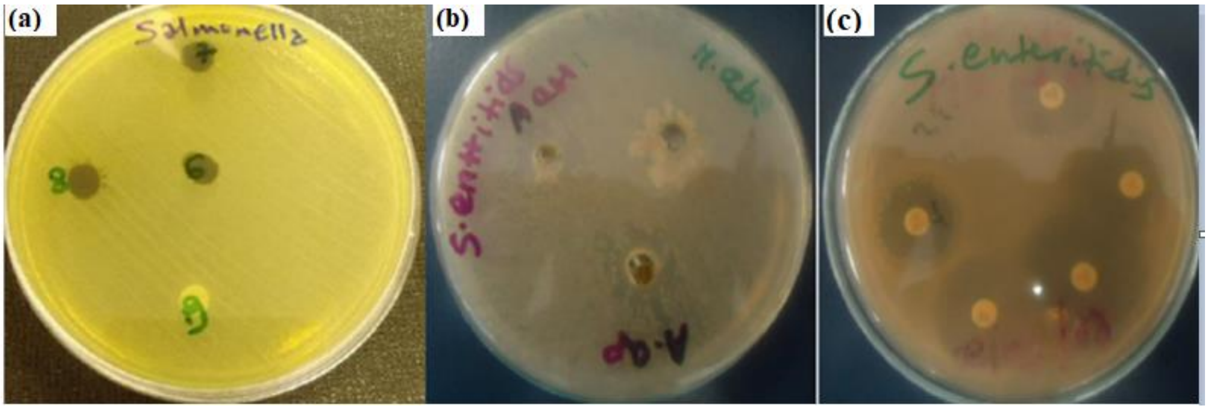

Fig. S2: (a) Zone of inhibition was detected for negative control. (b) Considerable amount of zone of inhibition was detected for test extract of Artemisia species especially with very clear zone of inhibition due to A.ap. (c) Acceptable zone of inhibitions were also detected for some impregnated antibiotics.
